# Telemedicine-Supported Intervention Versus Standard Care for Managing Cardiovascular Risk Factors in a Socially Deprived Urban Population: A Prospective Study

**DOI:** 10.3390/healthcare13172202

**Published:** 2025-09-03

**Authors:** Angelica Gherman, Codrina Mihaela Levai, Ovidiu Alin Haţegan, Călin Marius Popoiu, Emil Robert Stoicescu, Anca Laura Maghiari

**Affiliations:** 1 Ph.D. School Department, “Victor Babeş” University of Medicine and Pharmacy of Timisoara, 300041 Timisoara, Romania; angelica.gherman@umft.ro; 2 Research Center for Medical Communication, “Victor Babeş” University of Medicine and Pharmacy of Timisoara, Eftimie Murgu Square No. 2, 300041 Timisoara, Romania; codrinalevai@umft.ro; 3 Discipline of Medical Communications, Department 2—Microscopic Morphology, “Victor Babeş” University of Medicine and Pharmacy of Timisoara, 300041 Timisoara, Romania; 4 Discipline of Anatomy and Embriology, Medicine Faculty, Vasile Goldis Western University of Arad, Revolution Boulevard 94, 310025 Arad, Romania; 5 Department of Pediatric Surgery, “Victor Babeş” University of Medicine and Pharmacy of Timisoara, 300041 Timisoara, Romania; 6 Radiology and Medical Imaging University Clinic, “Victor Babeş” University of Medicine and Pharmacy of Timisoara, Eftimie Murgu Square No. 2, 300041 Timisoara, Romania; stoicescu.emil@umft.ro; 7 Research Center for Pharmaco-Toxicological Evaluations, “Victor Babeş” University of Medicine and Pharmacy of Timisoara, Eftimie Murgu Square No. 2, 300041 Timisoara, Romania; 8 Field of Applied Engineering Sciences, Specialization Statistical Methods and Techniques in Health and Clinical Research, Faculty of Mechanics, “Politehnica” University Timisoara, Mihai Viteazul Boulevard No. 1, 300222 Timisoara, Romania; 9 Department of Anatomy and Embriology, “Victor Babeş” University of Medicine and Pharmacy of Timisoara, 300041 Timișoara, Romania; boscu.anca@umft.ro

**Keywords:** cardiovascular risk factors, telemedicine, health disparities, blood pressure, cholesterol, fasting glucose, urban population, patient satisfaction

## Abstract

**Background:** Cardiovascular disease (CVD) remains a leading cause of morbidity and mortality, particularly in socioeconomically disadvantaged populations. Telemedicine offers a potential strategy to support risk factor management in such groups with limited access to care. Our aim was to assess the effectiveness of a telemedicine-supported intervention compared to usual care in improving cardiovascular risk parameters among adults from a socially deprived urban population. **Materials and Methods:** In this controlled intervention study, adult patients with one or more cardiovascular risk factors were recruited from a primary care center in a low-income urban neighborhood in Timişoara, Romania. Participants were allocated to either usual care or a six-month telemedicine-supported intervention group. The intervention consisted of regular phone calls by trained staff focusing on medication adherence, self-monitoring of blood pressure and glucose, smoking cessation, and lifestyle advice. No physical visits were delivered. Primary outcomes included changes in systolic and diastolic blood pressure, fasting glucose, and lipid profile. Data were collected at baseline and at six months. **Results:** A total of 144 patients were allocated to the telemedicine group and 142 to the usual care group. After 6 months, diastolic blood pressure decreased by 3.9 mmHg in the telemedicine group compared to 0.3 mmHg in the standard care group (*p* < 0.001). LDL-cholesterol was reduced by 18.0 mg/dL with telemedicine versus 5.7 mg/dL with usual care (*p* < 0.001). In contrast, fasting glucose improved more in the standard care group (–10.9 mg/dL vs. –2.0 mg/dL, *p* < 0.001). Patient satisfaction in the telemedicine group was high, with 84% rating the program as very useful. **Conclusions:** Basic telemedicine-supported interventions may represent a feasible and effective strategy for improving cardiovascular risk factors such as diastolic blood pressure and LDL-cholesterol in socially deprived populations. High satisfaction suggests strong acceptability; however, given the small sample size, short follow-up, and single-center design, these findings should be interpreted cautiously and confirmed in larger studies.

## 1. Introduction

Cardiovascular diseases (CVDs) are the leading cause of death globally, accounting for an estimated 17.7 million deaths in 2015, according to the World Health Organization, with a disproportionate burden falling on socially deprived populations [1,2,3]. Beyond mortality, CVD represents the most expensive health burden worldwide, with annual indirect costs projected to rise from USD 237 billion to USD 368 billion by 2035 [1]. Individuals living in underserved urban communities often face significant barriers to healthcare access, including transportation difficulties, limited health literacy, and strained healthcare infrastructure [4,5]. These challenges contribute to suboptimal management of cardiovascular risk factors, ultimately worsening health outcomes [6,7].

Cardiovascular risk factors comprise modifiable and non-modifiable variables, such as age, sex, elevated blood pressure, abnormal lipid profiles, and impaired glucose metabolism, that can collectively contribute to the development and clinical manifestation of CVD [8]. A sedentary lifestyle and the frequent intake of energy-dense, nutrient-poor foods high in saturated fats and sugars are also closely linked to the onset of atherosclerosis and metabolic disorders such as metabolic syndrome, diabetes mellitus, and hypertension, conditions that are highly prevalent in individuals with cardiovascular disease [9,10]. According to a recent investigation, behavioral factors have a greater impact on the management of cardiovascular diseases than preventative medicine [11,12]. Additionally, new research indicates that lowering certain risk factors, such as total cholesterol, arterial hypertension, and body mass index ( BMI ), may lower the death rate from cardiovascular disease [12,13]. Despite the fact that exercise and a balanced diet reduce the risk of CVD, heavy smokers who also followed these recommendations still had a 3.8-fold higher risk of CVD, which is consistent with previous research [12,14,15].

Telemedicine encompasses various modalities, including synchronous video consultations, asynchronous data exchange, remote patient monitoring, and mobile health applications, each tailored to enhance healthcare delivery and accessibility [16]. This field has emerged as a promising strategy to bridge gaps in care delivery, offering remote monitoring, counseling, and follow-up without the need for frequent in-person visits. While telemedicine interventions have shown efficacy in various clinical settings, evidence remains limited regarding their effectiveness in low-resource, high-risk urban populations [17,18,19]. It will be more important to assess the quality of care being provided and provide evidence that it is being maintained as telemedicine develops into a standard service in environments with limited resources [19]. In particular, the impact of basic, low-cost telemedicine solutions, as opposed to app-based or technologically advanced systems, requires further investigation to determine their feasibility and clinical utility in real-world primary care settings.

This study aimed to evaluate the effectiveness of a basic telemedicine intervention compared to usual care in improving cardiovascular risk profiles over a six-month period in a socially deprived urban population. The outcomes of interest included changes in blood pressure, lipid and glucose levels, BMI, and patient satisfaction, with the goal of informing scalable solutions to reduce health disparities.

## 2. Materials and Methods

### 2.1. Study Design

This was a prospective, quasi-experimental controlled study conducted in a primary care setting. Participants were consecutively enrolled during routine visits and allocated to either a telemedicine-supported intervention group or a standard care group. No randomization was performed; instead, allocation was structured to achieve comparable group sizes.

### 2.2. Population

This study targeted adults aged 40–80 years living in a socioeconomically deprived urban area of Timișoara, Romania. Eligible participants were those with at least one cardiovascular risk factor, defined as hypertension, dyslipidemia, impaired fasting glucose, or elevated BMI. Individuals with a known history of cardiovascular disease, severe psychiatric or cognitive disorders, or inability to provide informed consent were excluded.

Eligible participants were identified consecutively during routine visits at the primary care center. All eligible patients were informed about the study procedures and purpose, and those who agreed provided written informed consent. Enrollment continued until the planned sample size of 300 participants was reached.

Participants were allocated in equal numbers to the two study arms: 150 to the telemedicine-supported intervention group and 150 to the standard care group. Over the 6-month follow-up period, 286 participants (95.3%) completed the study, including 144 in the telemedicine arm and 142 in the standard care arm. Fourteen patients did not complete follow-up: in the telemedicine group, three moved away from the study area and three could not be contacted despite repeated attempts; in the standard care group, four moved away, three were lost to contact, and one withdrew consent. No adverse events related to the intervention were reported.

In the intervention group, telemedical care was delivered by trained general practitioners and nurses affiliated with the primary care center. Patients received monthly structured telephone consultations lasting approximately 15–20 min for six months. Each call followed a standardized checklist and included review of blood pressure and glucose self-monitoring logs, reinforcement of medication adherence, lifestyle counseling (dietary habits, physical activity, and smoking cessation), and reminders to complete laboratory evaluations. Nurses conducted most consultations under physician supervision, with cases requiring medical decision-making escalated to the supervising general practitioner.

The control group received standard care through local health services, consisting of usual clinic referrals and routine follow-up if patients chose to attend, without additional structured telephone support.

### 2.3. Data Collection

Data were collected at baseline and after six months for both groups. These assessments included measurements of systolic and diastolic blood pressure and BMI, laboratory analysis of fasting glucose and lipid profiles (total cholesterol, low-density lipoprotein (LDL) cholesterol, high-density lipoprotein (HDL) cholesterol, triglycerides), and a questionnaire capturing lifestyle behaviors and access to healthcare. Systolic and diastolic blood pressure were measured with validated automated sphygmomanometers (Omron M6, Omron Healthcare, Kyoto, Japan). Measurements were performed in a seated position, after 5 min of rest, on the right arm supported at heart level. Three readings were taken at one-minute intervals, and the average of the last two was recorded. Body weight and height were measured with participants wearing light clothing and no shoes, using calibrated scales and stadiometers. BMI was calculated as weight (kg)/height (m^2^). All laboratory tests were performed in accredited clinical laboratories. Venous blood samples were obtained after an overnight fast of at least 8 h. Standardized enzymatic methods were used for glucose and lipid profile (total cholesterol, LDL-c, HDL-c, triglycerides). In the intervention group, follow-up interviews were conducted at six months to assess patient satisfaction with telemedicine and perceived level of engagement, using a 5-point Likert scale questionnaire.

### 2.4. Outcomes

The primary outcomes were the changes from baseline to six months in systolic blood pressure (SBP), diastolic blood pressure (DBP), fasting glucose, and lipid levels. Secondary outcomes included changes in BMI, the proportion of patients who completed laboratory or primary care follow-up, self-reported lifestyle changes (improved diet or physical activity), and patient satisfaction within both groups.

### 2.5. Statistical Analysis

All analyses were conducted using IBM SPSS Statistics, version 29.0 (IBM Corp, Armonk, NY, USA) and Microsoft Excel for Microsoft 365 (Microsoft Corp., Redmont, WA, USA). Continuous variables were reported as means and standard deviations, and categorical variables were summarized using frequencies and percentages. Between-group comparisons at baseline and at six months were assessed using independent-sample t-tests for continuous variables and chi-square tests for categorical variables. Paired t-tests were used for within-group comparisons over time. To assess whether the change from baseline to 6 months differed significantly between the two groups, we calculated a *p*-value for interaction (‘*p*-value (difference in Δ from baseline)’), reflecting the difference in trajectories of the parameters across the 6-month period. To evaluate the magnitude of differences in changes between groups from baseline to six months, Cohen’s d effect sizes were calculated. Effect sizes were interpreted as small (0.2), moderate (0.5), or large (0.8). Achievement of therapeutic goals was assessed by comparing the proportion of patients in each group who met predefined metabolic targets at the 3-month follow-up. A two-sided *p*-value < 0.05 was considered statistically significant for all tests.

## 3. Results

### 3.1. Study Population

Out of 300 initially enrolled participants, 150 were allocated to the telemedicine group and 150 to the standard care group. Of these, 286 completed the study: 144 in the telemedicine arm and 142 in the standard care arm. The baseline characteristics of the two groups were generally comparable (Table 1). The mean age in the telemedicine group was 60.35 ± 7.47 years, while in the standard care group it was slightly higher at 62.38 ± 9.26 years, a difference that reached statistical significance (*p* = 0.03). The gender distribution was similar between groups, with women representing 53.5% in the telemedicine group and 45.8% in the standard care group (*p* = 0.55). No statistically significant differences were noted at baseline for the main cardiovascular and behavioral risk parameters (all *p*-values > 0.05), except for DBP (*p* = 0.02). The prevalence of hypertension (71.52% vs. 70.42%), diabetes mellitus (38.20% vs. 40.84%), and dyslipidemia (64.58% vs. 66.19%) was comparable across groups, with no statistically significant differences (all *p* > 0.60). Likewise, the proportions of patients receiving antihypertensive therapy (68.75% vs. 70.42%), lipid-lowering therapy (54.16% vs. 55.63%), and antidiabetic treatment (33.33% vs. 35.21%) were balanced between the telemedicine and standard care arms (all *p* > 0.70).

### 3.2. Six-Month Parameter Comparisons

At 6 months, improvements were noted across most parameters in both groups, but were significantly greater in the telemedicine group for DBP and LDL-cholesterol. However, improvements in BMI, SBP, triglycerides, and total cholesterol did not significantly differ between groups. Moreover, HDL-cholesterol and fasting glucose have shown important changes only in the standard care group. BMI decreased modestly in both groups, from 28.34 ± 3.66 to 27.52 ± 4.35 in the telemedicine group and from 27.57 ± 3.98 to 27.15 ± 4.60 in the standard care group. Although the reduction was slightly larger in the telemedicine group (mean change of −0.82 vs. −0.42), this difference was not statistically significant (*p* = 0.57). SBP declined by an average of 5.21 mmHg in the telemedicine group and 4.19 mmHg in the standard care group, with the between-group difference at 6 months approaching statistical significance (*p* = 0.08). DBP showed a statistically significant change in favor of the telemedicine group. The telemedicine group experienced a mean reduction of 3.93 mmHg, compared to a negligible change in the standard care group, where values slightly increased (88.63 ± 6.48 to 88.95 ± 6.59; *p* < 0.001). Fasting glucose improved more substantially in the standard care group, dropping from 125.31 ± 20.11 to 114.38 ± 17.91 mg/dL, with a mean reduction of 11.71 mg/dL. In contrast, the telemedicine group saw a smaller decrease from 126.09 ± 20.59 to 124.10 ± 19.01 mg/dL (−1.99 mg/dL), with a between-group *p*-value < 0.001. Total cholesterol decreased in both groups but to a slightly greater extent in the standard care arm (−14.27 mg/dL vs. −12.05 mg/dL in telemedicine). However, the *p*-value for between-group comparison at 6 months was not statistically significant (*p* = 0.20). HDL-cholesterol increased slightly in both groups (+3.01 mg/dL in telemedicine vs. +2.3 mg/dL in standard care), but the between-group difference did not reach statistical significance (*p* = 0.13). LDL-cholesterol saw a substantial reduction in the telemedicine group, from 136.03 ± 20.03 to 118.02 ± 18.05 mg/dL (−18.01 mg/dL), whereas in the standard care group, LDL dropped more modestly from 136.24 ± 20.96 to 130.54 ± 19.66 mg/dL (−5.70 mg/dL), with a *p*-value < 0.001. Triglycerides decreased by 3.95 mg/dL in the telemedicine group and slightly increased by 1.38 mg/dL in the standard care group (*p* = 0.07). Smoking status, dietary habits, and physical activity were obtained via self-reported questionnaires. These variables were not included among the primary clinical parameters due to potential reporting bias and subjectivity, in contrast to the objectively measured laboratory and anthropometric outcomes.

### 3.3. Achievement of Therapeutic Goals

Comparative analysis of cardiovascular and metabolic parameters revealed that patients in the telemedicine group were more likely to achieve therapeutic targets within the 3-month follow-up period than those receiving usual care.

For fasting glucose, however, the pattern was reversed: 66% of participants in the standard care group reached levels below 110 mg/dL by three months, compared to 43% in the telemedicine group (*p* < 0.01). In contrast, 71% of patients in the telemedicine group achieved systolic blood pressure values below 140 mmHg within the same timeframe, versus 66% in the standard care group. For diastolic blood pressure both groups showed almost the same achievement by the 3-month follow-up.

Lipid parameters followed a comparable pattern. In the telemedicine group, a greater proportion of patients reached total cholesterol levels below 200 mg/dL and LDL-cholesterol levels below 130 mg/dL by three months, compared to those in the usual care group. Triglyceride targets (<150 mg/dL) were also achieved more frequently among telemedicine patients during this period. These thresholds were used as pragmatic indicators of metabolic improvement, rather than guideline-based therapeutic targets that depend on individual cardiovascular risk categories.

### 3.4. Effect Sizes

**Figure 1:**
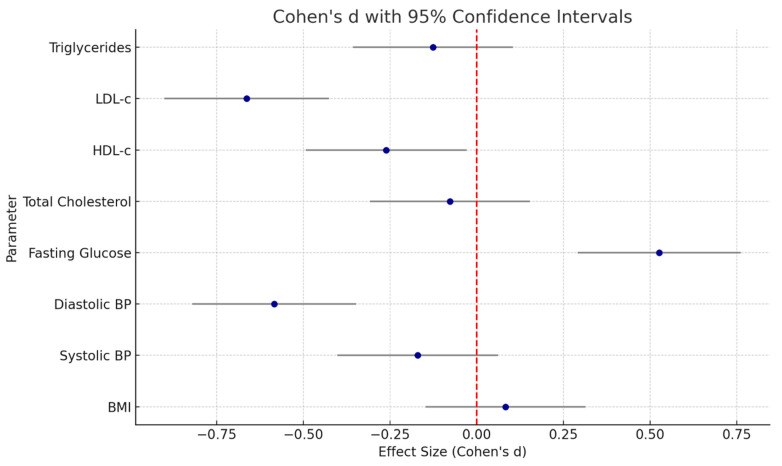
Forest plot of Cohen’s d effect sizes for cardiometabolic parameters.

The analysis of effect sizes for the change from baseline to six months between the telemedicine and standard care groups showed variable magnitudes across parameters, generally favoring the telemedicine group (Table 2, Figure 1).

LDL-cholesterol exhibited the largest effect (Cohen’s d = 0.68, 95% CI: 0.40 to 0.97), with a moderate-to-large benefit of telemedicine in reducing LDL levels compared to standard care. Fasting glucose also showed a moderate effect size (d = 0.53, 95% CI: 0.26 to 0.81). DBP demonstrated a similar moderate effect (d = 0.60, 95% CI: 0.32 to 0.88). In contrast, other parameters such as SBP, BMI, HDL-cholesterol, triglycerides, and total cholesterol had small or negligible effect sizes (d ranging from 0.01 to 0.27), and most of their confidence intervals crossed zero.

### 3.5. Satisfaction Results

Patient satisfaction was assessed at 6 months across several domains using a structured questionnaire with a 5-point Likert scale (1 = very unsatisfied, 5 = very satisfied) (Table 3). Results indicate a significantly higher satisfaction in the telemedicine group (mean = 4.2, SD = 0.6) compared to the usual care group (mean = 3.4, SD = 0.7). The difference was statistically significant (*p* < 0.001).

Patients reported greater accessibility (mean 4.6 ± 0.5 vs. 3.9 ± 0.7, *p* < 0.001), while time efficiency also favored telemedicine (4.5 ± 0.6 vs. 3.8 ± 0.8, *p* < 0.001). Similarly, comfort and convenience scored markedly higher in the telemedicine group (4.7 ± 0.5 vs. 4.0 ± 0.8, *p* < 0.001) However, some domains revealed a slight preference for in-person care. Usual care was rated higher in communication with the medical team (4.4 ± 0.6 vs. 4.2 ± 0.7, *p* = 0.034), and trust in the care provider (4.5 ± 0.5 vs. 4.3 ± 0.6, *p* = 0.031).

No statistically significant difference was observed in patients’ understanding of their treatment plans (4.4 ± 0.6 vs. 4.3 ± 0.6, *p* = 0.217), indicating that both care models communicated therapeutic goals effectively. Additionally, technical ease of use received a high average score of 4.5 ± 0.5 in the telemedicine group. Overall, the results support the feasibility and acceptability of telemedicine in delivering patient-centered care, while also highlighting areas where in-person care may retain an advantage.

### 3.6. Dropout Rate and Adherence

The study completion rate was high in both arms, with only minor dropout due to relocation or loss of contact (14 patients in total, 6 patients from the telemedicine group and 8 patients from the standard care group). No adverse events related to the telemedicine intervention were reported.

Adherence to lifestyle recommendations was self-reported to be higher in the telemedicine group during interim follow-up calls. At 6 months, a greater proportion of participants in the telemedicine group reported adopting healthier behaviors, with some differences reaching statistical significance. Improved dietary habits were reported by 79.8% of individuals in the telemedicine group, compared to 63.4% in the usual care group (*p* = 0.001). Increased physical activity, defined as engaging in at least 150 min of moderate activity per week, was reported by 52.1% of telemedicine participants and 54.9% of those in the control group (*p* = 0.71), showing no significant difference. Smoking prevalence remained relatively stable in both groups, with 22.2% of participants in the telemedicine group and 23.2% in the usual care group reporting current smoking at 6 months (*p* = 0.94). However, only the change in reported dietary habits showed a statistically significant difference over time between groups (*p* for interaction = 0.001), while changes in smoking and physical activity did not (*p* for interaction = 1.0 and 0.98, respectively).

## 4. Discussion

This study was carried out in a socially disadvantaged urban community with little access to digital technology. We addressed the often-overlooked digital divide by deliberately avoiding reliance on smartphone apps and instead using simple telephone-based telemedicine. Through familiar and easily available communication techniques, participants who could have been excluded from traditional mHealth initiatives were actively engaged. Low-tech telemedicine models have the potential to overcome healthcare gaps in underprivileged communities, as seen by the better clinical outcomes and increased satisfaction seen in the telemedicine group. This strategy could be modified for wider application in comparable high-risk, resource-constrained contexts due to its simplicity and scalability.

The study demonstrates that a structured, low-tech telemedicine intervention significantly improved multiple cardiovascular risk factors in a socially vulnerable adult population over a six-month period. Compared with standard care, the telemedicine group achieved more pronounced reductions in systolic and diastolic blood pressure, LDL-cholesterol, and triglycerides, with moderate-to-large effect sizes.

Our results align with findings from previous randomized trials and meta-analyses evaluating digital health interventions in chronic disease management. Other telemedicine-based interventions demonstrated overall beneficial effects on key cardiovascular risk factors, yielding small but statistically significant reductions in HbA1c among patients with diabetes (g = −0.432, *p* < 0.001), moderate reductions in systolic blood pressure (g = −0.775, *p* < 0.001), small reductions in diastolic blood pressure (g = −0.447, *p* < 0.001), and moderate reductions in body weight among overweight individuals (g = −0.628, *p* < 0.001) [20]. Pogosova et al. showed that the intervention group showed significantly greater reductions in weight, waist circumference, BMI, diastolic blood pressure, total and LDL-cholesterol, and body fat mass compared to controls. Lean mass was higher, and both anxiety and depression scores were significantly lower in the intervention group [21]. A meta-analysis by Omboni et al. reported that telemonitoring and teleconsultations significantly reduced both systolic and diastolic blood pressure, with average reductions of 4.7 mmHg and 2.5 mmHg, respectively, changes comparable to our observed improvements in SBP and DBP [22]. Another study exhibited no significant changes in BMI, blood pressure (both systolic and diastolic), or lipid profiles, including total cholesterol, HDL-c, LDL-c, and triglycerides, in either hospital-based or home-based cardiac rehabilitation programs. These parameters remained largely stable from baseline to post-intervention, with *p*-values > 0.05 for both within-group and between-group comparisons [23]. A follow-up study of an mHealth intervention involving motivational calls and SMS found no significant impact on blood pressure after 5 years, though a trend toward reduced hypertension risk was noted. However, meaningful reductions in body weight (−5.42 kg) and BMI (−2.56 kg/m^2^) were observed, especially among those who received at least half of the scheduled calls. Unlike our study, these changes in BMI and weight became evident due to the longer follow-up period of 5 years [24]. Our observed reductions in fasting glucose levels are different from the findings of the TELESAGE study, where patients receiving remote diabetes management achieved better glycemic control and treatment adherence than those in usual care [25]. Similarly, a telemedicine-supported lifestyle intervention led to a modest but statistically significant reduction in HbA1c at 6 months compared to usual care (−0.13%, 95% CI −0.25 to −0.01; *p* = 0.04). However, this effect was not maintained at 12 months once individualized feedback and health literacy support were discontinued [26].

Compared to high-tech platforms relying on apps or wearables, our study used only monthly phone calls conducted by nurses or general practitioners. This approach not only achieved measurable health improvements, but did so among patients in a socially deprived urban area, a population often overlooked in digital health research. Similar low-tech interventions have shown efficacy in India, sub-Saharan Africa, and Latin America, where phone-based follow-up improved blood pressure and glycemic outcomes in hypertensive or diabetic patients [27,28,29]. However, few of these studies have included a broad set of cardiometabolic outcomes or stratified by socioeconomic vulnerability.

Notably, the improvements in LDL-cholesterol in our telemedicine group, although modest in magnitude, suggest favorable lipid modulation through lifestyle and medication adherence. In contrast to our findings, some previous studies have reported minimal effects of telemedicine on lipid profiles. For example, a trial by Margolis et al. observed no significant differences in LDL-c at 6 months, suggesting that results may vary depending on patient engagement, baseline risk, and the intensity of the intervention. A meta-analysis of 54 studies found that telemedicine interventions modestly reduced blood pressure and HbA1c levels, while effects on lipid profiles and BMI were inconsistent and clinically insignificant [30]. Our more pronounced improvement in triglycerides (−3.95 mg/dL) and total cholesterol (−12.05 mg/dL) may reflect the combined effect of dietary counseling, physical activity encouragement, and regular feedback via phone.

In a trial by Garcia et al., lipid levels improved significantly after the one-year follow-up. LDL-c decreased from 108.4 ± 40.6 to 48.7 ± 14.4 mg/dL. The proportions of patients achieving LDL-c <100, <70, and <55 mg/dL increased from 44.5%, 17.6%, and 7.2% at baseline to 100%, 95.4%, and 81.5% at study end [31].

The speed of achieving clinical targets was also superior in the telemedicine group. These findings suggest that structured support may facilitate attainment of blood pressure and lipid targets through improved adherence and monitoring. However, this effect did not extend to glycemic control, where patients in the standard care group achieved fasting glucose targets more frequently. Possible explanations include differences in medication use, variability in self-monitoring, and the relatively short follow-up period. A study from the United States evaluated changes in HbA1c among patients with poorly controlled type 2 diabetes (HbA1c > 8%) enrolled in a pharmacy-driven telehealth program. The analysis compared glycemic outcomes between two time periods: pre-COVID-19 and during COVID-19. After 3 months, the COVID-19 group showed a mean HbA1c reduction of 2.0%, compared to 1.3% in the pre-COVID group (*p* = 0.305); at 6 months, the reductions were 2.2% vs. 1.2%, respectively (*p* = 0.249) [32]. Another study reported after just three months of intervention that patients using the cellular phone-based telemedicine system showed a significantly greater reduction in HbA1c levels compared to those using standard self-monitoring. While the control group experienced a modest and non-significant decrease of 0.20%, the telemedicine group achieved a meaningful drop of 0.61% (*p* < 0.001), with even greater improvement (0.81%) among those starting with HbA1c ≥ 8% [33].

Patient satisfaction was another key strength of the telemedicine arm. Higher scores in accessibility, convenience, and perceived effectiveness are consistent with large-scale surveys conducted during the COVID-19 pandemic, where telemedicine was broadly endorsed for chronic disease follow-up [34]. Importantly, trust and provider communication were slightly favored in the usual care group, echoing the concern that some aspects of rapport may be diluted in remote settings—a limitation that should be addressed in future hybrid models of care. However, another study showed that among 1026 respondents, 71% preferred in-person visits and 29% preferred telemedicine, exposing a significant difference (*p* < 0.0001). Overall, telemedicine was seen as convenient and accessible, especially for chronic or mobility-limited patients, but in-person care remained the preferred choice for most [35]. In another research work involving large-sized sample groups, patient satisfaction with telemedicine was similar to in-person visits, with early lower scores for clinical communication improving over time. Telemedicine was rated higher for appointment access early in the pandemic, but in-person visits were slightly preferred for doctor interaction and care explanations [36].

Despite these promising outcomes, several limitations merit consideration. First, although random allocation was used, full blinding was not possible due to the nature of the intervention, which could introduce bias in patient-reported outcomes. Second, self-reported lifestyle improvements may overestimate behavioral change. Third, the duration of follow-up (6 months) is relatively short, and long-term sustainability of benefits remains uncertain.

In summary, this study provides compelling evidence that structured, low-tech telemedicine can effectively reduce cardiovascular risk factors in disadvantaged populations. The observed improvements, comparable to or exceeding those seen in more technologically complex interventions, show the importance of inclusivity and simplicity in digital health strategies. Our findings support broader implementation of basic telemedicine tools, not as replacements for in-person care, but as scalable complements that can help bridge health equity gaps in cardiovascular prevention.

## 5. Conclusions

This study suggests that a simple, low-cost telemedicine intervention may help improve selected cardiovascular and metabolic outcomes in a socially deprived urban population. Patients receiving structured phone-based support achieved better control of diastolic blood pressure and LDL-cholesterol and were more likely to reach treatment targets within three months. However, patients from the standard care group reached better fasting glucose levels. The intervention was well accepted, with high satisfaction and good adherence. Taken together, these findings support the feasibility and acceptability of low-tech telemedicine as a potentially scalable strategy to improve care in underserved settings. Nonetheless, the results should be interpreted with caution due to the relatively small sample size, short follow-up period, and single-center design, and confirmation in larger, multicenter trials is warranted.

## Figures and Tables

**Table 1:** Baseline clinical parameter comparison between telemedicine and standard care groups.

Parameter	Telemedicine (Baseline)	Standard Care (Baseline)	*p*-Value (Baseline)	Telemedicine (6 Months)	Standard Care (6 Months)	*p*-Value (6 Months)	*p*-Value (Difference in Δ from Baseline)
Age (years)	60.35 ± 7.47	62.38 ± 9.26	0.03	–	–	–	–
Gender (male/female, %)	46.5/53.5	54.2/45.8	0.55	–	–	–	–
Smoking	21.5%	23.2%	0.83	22.2%	23.2%	0.94	1
Physically active (%)	48.6%	50.7%	0.81	52.08%	54.92%	0.71	0.98
Healthy diet reported (%)	61.8%	58.4%	0.64	79.83%	63.38%	0.003	0.001
BMI (kg/m^2^)	28.34 ± 3.66	27.57 ± 3.98	0.21	27.52 ± 4.35	27.15 ± 4.60	0.65	0.57
Systolic BP (mmHg)	141.23 ± 12.49	142.24 ± 13.75	0.27	136.02 ± 11.09	138.05 ± 12.72	0.16	0.08
Diastolic BP (mmHg)	88.99 ± 7.48	88.63 ± 6.48	0.02	85.06 ± 6.73	88.95 ± 6.59	<0.001	<0.001
Fasting glucose (mg/dL)	126.09 ± 20.59	125.31 ± 20.11	0.74	124.10 ± 19.01	114.38 ± 17.91	<0.001	<0.001
Total cholesterol (mg/dL)	161.76 ± 37.85	167.26 ± 41.99	0.24	149.71 ± 39.57	152.99 ± 45.01	0.27	0.20
HDL-c (mg/dL)	46.86 ± 7.71	46.18 ± 8.39	0.66	46.50 ± 7.80	48.48 ± 7.34	0.03	0.13
LDL-c (mg/dL)	136.03 ± 20.03	136.24 ± 20.96	0.21	118.02 ± 18.05	130.54 ± 19.66	<0.001	<0.001
Triglycerides (mg/dL)	166.41 ± 41.34	165.83 ± 38.12	0.90	162.46 ± 33.74	167.21 ± 41.27	0.28	0.07

Abbreviations: BMI—body mass index; BP—blood pressure; HDL—high-density lipoprotein; LDL—low-density lipoprotein. The last column indicates whether the change from baseline to 6 months differed significantly between telemedicine and standard care.

**Table 2:** Cohen’s d effect sizes for cardiometabolic measures.

Parameter	Cohen’s *d* (95% CI)
BMI	0.08 (−0.14 to 0.31)
Systolic BP	−0.17 (−0.40 to 0.06)
Diastolic BP	−0.58 (−0.82 to −0.34)
Fasting Glucose	0.52 (0.29 to 0.76)
Total Cholesterol	−0.07 (−0.30 to 0.15)
HDL-c	−0.26 (−0.49 to −0.02)
LDL-c	−0.66 (−0.90 to −0.42)
Triglycerides	−0.12 (−0.35 to 0.10)

**Table 3:** Patient satisfaction ratings across groups.

Domain	Telemedicine (Mean ± SD)	Standard Care (Mean ± SD)	*p*-Value
Accessibility of care	4.6 ± 0.5	3.9 ± 0.7	<0.001
Time efficiency	4.5 ± 0.6	3.8 ± 0.8	<0.001
Communication with medical team	4.2 ± 0.7	4.4 ± 0.6	0.03
Comfort and convenience	4.7 ± 0.5	4.0 ± 0.8	<0.001
Understanding of treatment plan	4.4 ± 0.6	4.3 ± 0.6	0.21
Perceived effectiveness	4.5 ± 0.6	4.2 ± 0.7	0.006
Trust in care provider	4.3 ± 0.6	4.5 ± 0.5	0.03
Technical ease of use	4.5 ± 0.5	—	—

## Data Availability

The data presented in this study are available on request from the corresponding author due to privacy and ethical restrictions.

## Abbreviations

The following abbreviations are used in this manuscript:

BMI	body mass index
COVID-19	coronavirus disease 2019
CVD	cardiovascular disease
DBP	diastolic blood pressure
HDL	high-density lipoprotein
LDL	low-density lipoprotein
SBP	systolic blood pressure
